# A KDM4A-PAF1-mediated epigenomic network is essential for acute myeloid leukemia cell self-renewal and survival

**DOI:** 10.1038/s41419-021-03738-0

**Published:** 2021-06-03

**Authors:** Matthew E. Massett, Laura Monaghan, Shaun Patterson, Niamh Mannion, Roderick P. Bunschoten, Alex Hoose, Sandra Marmiroli, Robert M. J. Liskamp, Heather G. Jørgensen, David Vetrie, Alison M. Michie, Xu Huang

**Affiliations:** 1 Paul O’Gorman Leukaemia Research Centre, Institute of Cancer Sciences, University of Glasgow, Glasgow, United Kingdom; 2 Medicinal Chemistry Department, Joseph Black Building, School of Chemistry, University of Glasgow, Glasgow, United Kingdom; 3 Cellular Signalling Unit, Department of Biomedical, Metabolic and Neural Sciences, University of Modena and Reggio Emilia, Modena, 41125 Italy; 4 Wolfson Wohl Cancer Research Centre, Institute of Cancer Sciences, University of Glasgow, Glasgow, United Kingdom

**Keywords:** Cancer genomics, Apoptosis

## Abstract

Epigenomic dysregulation is a common pathological feature in human hematological malignancies. H3K9me3 emerges as an important epigenomic marker in acute myeloid leukemia (AML). Its associated methyltransferases, such as SETDB1, suppress AML leukemogenesis, whilst H3K9me3 demethylases KDM4C is required for mixed-lineage leukemia rearranged AML. However, the specific role and molecular mechanism of action of another member of the KDM4 family, KDM4A has not previously been clearly defined. In this study, we delineated and functionally validated the epigenomic network regulated by KDM4A. We show that selective loss of KDM4A is sufficient to induce apoptosis in a broad spectrum of human AML cells. This detrimental phenotype results from a global accumulation of H3K9me3 and H3K27me3 at KDM4A targeted genomic loci thereby causing downregulation of a *KDM4A*-*PAF1* controlled transcriptional program essential for leukemogenesis, distinct from that of KDM4C. From this regulatory network, we further extracted a *KDM4A-9* gene signature enriched with leukemia stem cell activity; the *KDM4A-9* score alone or in combination with the known *LSC1*7 score, effectively stratifies high-risk AML patients. Together, these results establish the essential and unique role of KDM4A for AML self-renewal and survival, supporting further investigation of KDM4A and its targets as a potential therapeutic vulnerability in AML.

## Introduction

Epigenetic regulators are frequently mutated in acute myeloid leukemia (AML), leading to epigenomic alterations^1^. Inhibitors that target epigenetic modulators to rectify epigenomic abnormalities represent valid therapeutic strategies. Further understanding of how epigenetic dysregulation in AML contributes to leukemogenesis may uncover tractable therapeutic targets and biomarkers for AML patient treatment and/or prognostic evaluation.

Polymerase-associated factor 1 (PAF1), a core component of the PAF complex (PAFc) is essential in AML^2–4^. PAFc mediated recruitment of the H3K9 methyltransferases, such as SETDB1 antagonizes Mixed-Lineage Leukemia (MLL) signaling, and inhibition of SETB1 promotes AML cell proliferation^5,6^.

Primary AML blasts from patients with poor prognosis feature global H3K9me3 hypomethylation^7^ positing an oncogenic role for H3K9me3 demethylases in AML. Cheung et al. identified an H3K9me3 demethylase, KDM4C as a cofactor of the PRMT1 complex in MLL rearranged (MLLr) and MOZ-TIF2 AML^8^. Simultaneous knockout (KO) of all three members of the Kdm4 family (*kdm4a/b/c)* in mice attenuates MLL-AF9 AML^9^, indicating roles for the Kdm4 family in murine myeloid leukemia. However, the therapeutic benefit of targeting the KDM4 family in human AML is not well understood.

Our previous lentiviral knockdown (KD) screen targeting epigenetic regulators in 12 human AML cell lines representing several AML subgroups found that KMD4A KD leads to significant suppression of leukemia cell proliferation^10^. KDM4A has different roles in normal tissue development compared to other members of the KDM4 family; it is amplified/overexpressed in various malignancies including AML (Fig. S1A) and correlates with poor outcome in ovarian cancer^11,12^. Herein we demonstrate that KDM4A KD induces AML apoptosis by a unique mechanism to KDM4C in myeloid leukemia. Apoptosis results from a global accumulation of H3K9me3 and H3K27me3 at KDM4A genomic loci thereby causing downregulation of a *KDM4A*-*PAF1-*mediated oncogenic program, including a 9-gene signature enriched with leukemia stem cell (LSC) activity, which can stratify high-risk patients. These findings support an essential and unique role of KDM4A for AML cell self-renewal and survival.

## Materials and methods

### Reagents, plasmids, and virus manufacture

Puromycin, IOX1^dev^, and lentiviral constructs for KD experiments (Supplemental Table) were purchased from Sigma-Aldrich (St. Louis, MO, USA). IOX1 was from Tocris (4464). pLenti-HA-KDM4A wt and mut (H188A/E190A) were a gift from Dr. Gary Spencer (CRUK Manchester Institute). Lentiviral and retroviral supernatants were prepared, and leukemic human and murine cells transduced with viral particles as previously described^10^. A list of antibodies used in flow cytometry and Western blot/immunoprecipitation and immunofluorescence staining is in the Supplemental Table.

### Culture of cell lines and primary cells

Leukemia cell lines were from DMSZ (Braunschweig, Germany) and grown in RPMI (10% FBS and 2 mM L-Glutamine) at 37 °C in 5% CO_2_. They were recently authenticated and tested for mycoplasma contamination. Murine and human primary AML and normal BM samples were cultured in serum-free media (SFM) (H5100, Stem Cell Technologies, UK) supplemented with appropriate cytokines as described^13^. Murine MLL-AF9 AML cells were leukemic BM cells extracted from a mice cohort with MLL-AF9 AML established by Somervaille et al.^14^, and cultured in the conditional medium with mIL-3 (100 ng/ml). Cytokines purchased from PeproTech (London, UK).

### Cell proliferation, apoptosis, and cell cycle analysis

Cell viability was measured by cell count using trypan blue dye (Sigma, T8154) /hemocytometer or resazurin (Alamarblue dye, Sigma) with the Envision Fluorescent Reader (Perkin Elmer). Apoptosis was assessed using Annexin-V/Dead Cell Apoptosis Kit (V13241, ThermoFisher) and cell cycle analysis with PI/RNase Staining solution (F10797, ThermoFisher) as per manufactures’ instructions. Data were acquired using an LSRII flow cytometer and an Aria III flow cytometer (BD Biosciences, UK) and analyzed using FlowJo software (Tree Star Inc., USA).

### Protein extraction and Western blot

Cell pellets were suspended in fresh lysis buffer (50 mM Tris-HCl, pH7.4, 150 mM NaCl, 1% Triton X-100, 0.5% Sodium Deoxycholate, 0.5% SDS, 0.1% β-mercaptoethanol, 10 mM Sodium butyrate, 10 mM nicotinamide, 1 mM PMSF, 1× phosphatase inhibitor, 1× protease inhibitor) at 30 μl per 10^6^ cells and lysed on ice. Protein Lysates were quantified using Pierce Coomassie Plus Bradford and ran in NuPAGE™ 4–12% Bis-Tris Protein Gels (Invitrogen) as per products’ instructions. Only one loading control is used when Western blot analyses with multiple antibodies have been done sequentially in the same membrane.

### Immunofluorescence (IF) staining

6 × 10^4^ cells per condition were incubated on poly-L-lysine coated Hendley-Essex 12 well glass microscope slides for 1 h before being fixed in 4% formaldehyde in PBS. The cells were permeabilized in 0.5% Triton-X-100 PBS followed by 2 h of blocking in 5% BSA, 0.2% Triton-X-100 TBS. Primary antibody was applied overnight in a humidified chamber at 4 °C. Appropriate secondary antibody (1:500 dilution) was applied for further 1 h incubation at room temperature after removal of a primary antibody using PBS 0.1% Tween 20. Antifade mountant with DAPI reagent (Thermo Fisher #P36962) was used to seal each sample and images were captured on the Zeiss Axioimager M1 Epifluorescence and Brightfield Microscope. CellProfiler v2.2.0 image analysis software (CellProfiler) was used to quantify IF signals.

### Colony-forming cell assay

Colony-forming cell (CFC) assay for murine cells was performed by plating 1000 cells on methylcellulose (MethoCult M3434, Stem Cell Technologies). Colony Assay for human CD34^+^ hematopoietic stem and progenitor cells (HSPC) and AML patient cells were performed by plating 10000 cells and 3000 cells, respectively, on methylcellulose (MethoCult H4434, Stem Cell Technologies). CFU-GM (Granulocyte/Macrophage), M(Macrophage), and E(Erythroid) colonies were enumerated 10 days after seeding.

### Murine transplantation experiments

Mice experiments were approved by the local animal ethics board and performed under a project license issued by the United Kingdom Home Office, in keeping with the Home Office Animal Scientific Procedures Act, 1986. Non-obese diabetic. Cg-Prkdc scid Il2rgtm1Wjl/SzJ (NSG) mice were purchased from Jackson Laboratories (Bar Harbor, ME, USA) for transplantation as previously described^10^. Primary AML patient samples for xenograft transplantation were unfractioned primary blasts from our and Manchester biobank collections. Control or *KDM4A* KD human AML THP1 cells or primary AML patient blasts were FACS sorted 40 h following lentiviral infection and immediately transplanted into sub-lethally irradiated (450 cGy) NSG mice of 6–8-week-old, mixed-sex (10,000 THP1 cells or 10^6 primary AML cells) via tail vein injection.

### RNA isolation, quantitative PCR, RNA-seq, and ChIP-seq

RNA was extracted using QIAshredder™ columns and RNeasy Plus Microkit™ (Qiagen). RNA-seq libraries were produced using the TruSeq® stranded mRNA kit (Illumina) and sequenced on the Illumina NextSeq™ 500 platform. For ChIP-seq, DNA was purified using Diagenode’s iPure kitv2 and libraries made using the TruSeq ChIP Library Preparation Kit according to the manufacturer’s instructions. For QPCR, reverse transcription was carried out using Invitrogen SuperScript III First-Strand Synthesis kit. A SYBR^®^ green-based fluorescent system was used to quantify dsDNA using the Applied Biosystems 7900 HT Fast Real-Time PCR system. Each qPCR plate included technical triplicates of each specific target alongside two housekeeping genes (GAPDH and ß-Actin). Delta-Delta CT method was used for analysis of gene expression against control. RNA-seq and ChIP-seq reads were mapped to the hg19 human genome or mm9. Transcript abundances were calculated in transcripts per million (TPM) using Kallisto^15^. SICER was used for peak calling on default settings. Both data files are available in the Gene Expression Omnibus (GEO): GSE125376.

### Gene signature construction

The *KDM4A-9* signature score is calculated as shown below using the least absolute shrinkage and selection operator (LASSO) linear regression^16,17^.

$$KDM4A - 9\,{\mathrm{signature}}\,{\mathrm{score}} = \mathop {\sum}\limits_{n = 1}^9 {x_i\,r_i}$$

Where *x*_i_ and *r*_i_ denote the gene expression and coefficient of the *i*th gene (out of the total 9 genes) in the signature, respectively. Specifically, *KDM4A-9* score = (*TPM2* × 0.097292) + (*CD82* × 0.038719) + (*SLC29A2* × 0.167487) + (*INF2* × 0.150025) + (*STAR* × −0.13354) + (*ACP6* × 0.04633) + (*IFI6* × 0.015753) + (*MROH6* × 0.086738) + (*GSDMD* × 0.008289).

### Correlating the *KDM4A-9* signature and LSC activity

LSC enrichment classification and raw expression data from AML samples (GSE76008)^17^ were analyzed using the lumi 2.36.0 package. Intensity values were normalized by Robust spline normalization. The diagnostic capability of each gene signature to predict LSC activity across AML samples (GSE76008) was assessed by ROC (Receiver operating characteristic) curve analysis. The Youden index was used to identify the optimal cut-off value.

### Construction of *KDM4A-9* and *LSC17* combined score

Min-Max scaling of the *KDM4A-9* and *LSC17* scores was performed prior to the linear summation of *KDM4A-9* and *LSC17* scores for each patient to generate a combined *KDM4A-9/LSC17* score.

$${\mathrm{X}}_{{\mathrm{new}}} = \frac{{{\mathrm{X}}_{\mathrm{i}} - \min \left( {\mathrm{X}} \right)}}{{\max \left( {\mathrm{X}} \right) - \min \left( {\mathrm{X}} \right)}}$$

### Network construction and visualization

To visualize the connections between the *LSC17* and *KDM4A-9* signature genes, as well as *KDM4A* and *PAF1* in AML patients, a filtered edge list (TOM ≥ 0.05)^18^ was constructed. An undirected network graph was generated using the graph.edgelist function from the igraph 1.2.5 package.

### Motif analysis

Motif enrichment analysis of 400 bp length DNA sequences centered over the TSS of KDM4A or PAF1 bound (FDR ≤ 0.01) and DE (padj ≤ 0.05, DE ≥ 0.5 or ≤−0.5) genes was performed using MEME-ChIP (http://meme-suite.org/tools/meme-chip).

### Statistical analysis

Normally distributed groups were compared using a two-tailed student *t*-test unless stated otherwise. Survival probabilities estimated by Kaplan-Meier method. For RNA-seq counts, a pseudo-count of 1 was added prior to log_2_-transformation. Statistical significance of differential gene expression was assessed by Welch’s *t*-test unless otherwise stated. For RNA-seq, differential expression analysis was performed using the DESeq2 1.26.0 R package. Statistics were calculated using R-3.6.1.

## Results

### KDM4A is required for the survival of human and murine AML cells

**Fig. 1:**
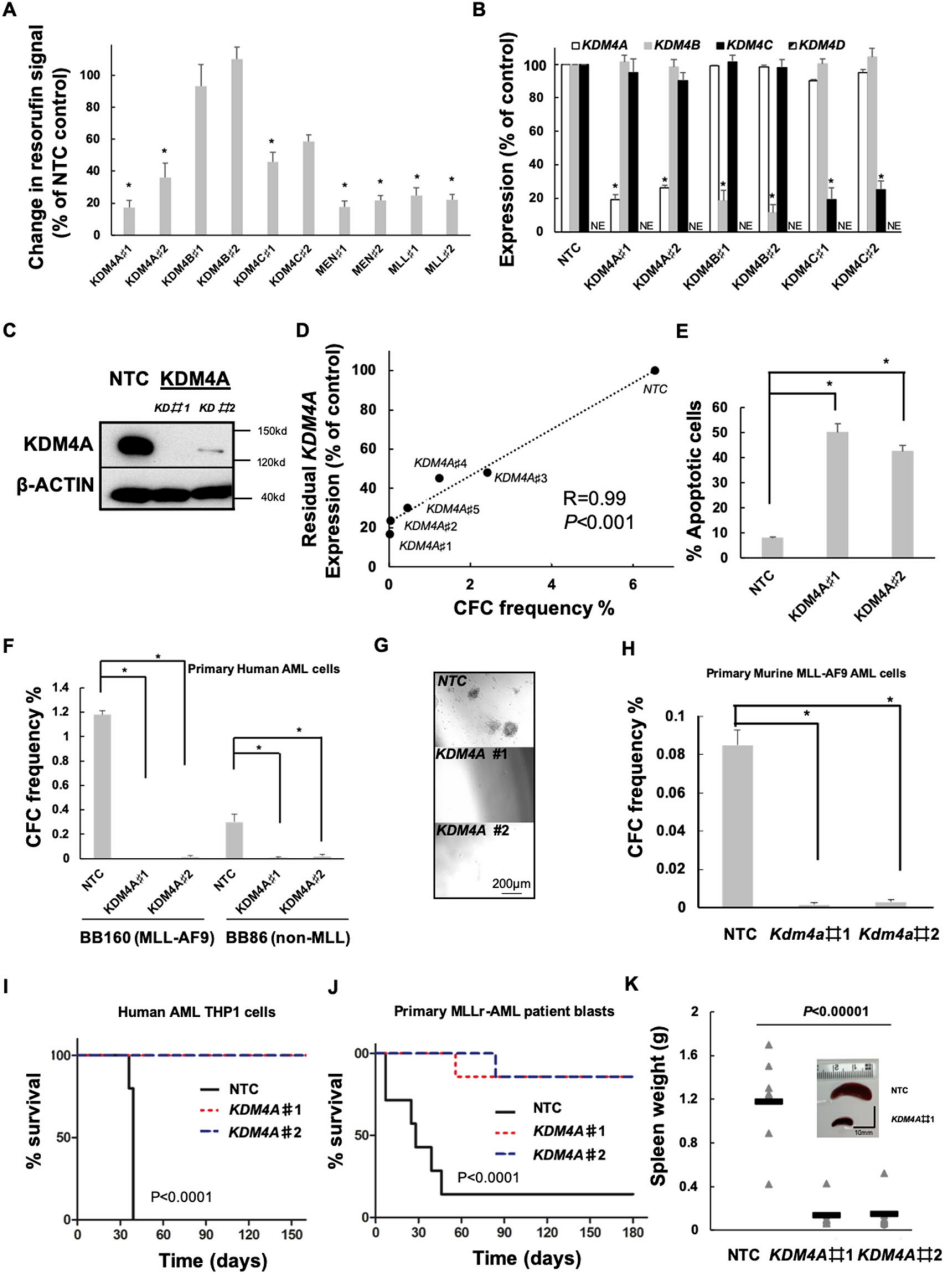
KDM4A is required for the functional potential of human and murine AML cells. **A**–**E** Human THP1 AML cells were transduced with lentiviruses targeting *KDM4A* or other KDM4 family members for KD (♯1 and ♯2 represent individual distinct lentiviruses targeting genes for KD as indicated), or a non-targeting control (NTC), using KD of *MLL* and *MEN1*, as positive controls, which are essential genes for AML cell proliferation. All bar charts show mean ± s.e.m. **A** Resorufin signal after 4 days of individual KDM4 family member KD relative to NTC control cells (*n* = 3); *p < 0.01 for comparison of each KD versus *NTC*. **B** Expression of *KDM4A/B/C/D* in indicated KD cells relative to NTC control cells (*n* = 3); *p < 0.001. **C** Representative immunoblot showing *KDM4A* KD in THP1 cells (*n* = 3). **D** Scatter plot shows the correlation of *KDM4A* KD with inhibition of frequency of colony-forming cells (CFC) enumerated following 10 days in semisolid culture (*n* = 3), as determined by QPCR; *p < 0.001. **E** Percentage of apoptotic cells determined by Annexin V^+^/ 7AAD^+/−^ staining on day 4 of liquid culture after puromycin selection (*n* = 3); *p < 0.001. **F**–**G** The indicated primary unfractioned patient blasts were transduced with lentiviruses targeting *KDM4A* for KD, or an NTC. Primary AML cells used include BB160, containing t(9;11) (MLL-AF9) chromosomal translocation and BB86 (normal cytogenetics, non-MLL) (BB number is the Manchester Cancer Research Centre Biobank sample identifier). All bar charts show mean ± s.e.m. **F** CFC frequencies of primary human AML blasts (*n* = 3) following lentivirus infection, puromycin selection, and initiation of *KDM4A* KD; *p < 0.0001. **G** Representative images from **F**. **H** CFC frequencies of primary murine MLL-AF9 AML cells following *KDM4A* depletion (*n* = 3); *p < 0.0001. **I** Survival curves of NSG mice transplanted with 10,000 *KDM4A* KD or NTC THP1 cells (*n* = 5 per cohort); *p* by log-rank test. **J** Survival curves of NSG mice transplanted with 10^6^ *KDM4A* KD or NTC primary AML cells (BB160, *n* = 7 per cohort); *p* by log-rank test. **K** Spleen weights of mice from **J** with a re*p*resentative image of the spleen. *p* by one-way ANOVA, *F* = 34.13045.

*KDM4A* expression is unique (Fig. S1B) being highly enriched in AML-LSC^+^ populations (Fig. S1C), suggesting that KDM4A is important for LSC, which are negatively correlated with AML patient survival. We performed lentivirus shRNA KD of *KDM4A*, *KDM4B*, and *KDM4C* in human AML MLL-AF9-driven THP1 cells to confirm its essential role. *KDM4A* KD THP1 cells exhibited the greatest decrease in cell proliferation compared to non-targeting cells (NTC) (Fig. 1A–C). Consistent with previous work, lentiviral KD of *KDM4C* had an inhibitory effect on cell proliferation^8^ (Fig. 1A). CFC potential was positively correlated with the *KDM4A* in a dose-dependent manner when five *KDM4A* KD shRNA targeting constructs were compared (Fig. 1D). *KDM4A* KD induced apoptosis (Fig. 1E and S1D) rather than cell cycle arrest (Fig. S1E). These results were further confirmed in primary patient blasts (Fig. 1F and G) and murine AML cells (Fig. 1H). Importantly, we determined the impact of *KDM4A* KD on AML initiation in vivo by transplanting *KDM4A* KD THP1 cells (Fig. 1I) or primary AML cells (Fig. 1J–K and S1F–S1H) into recipient NSG mice. Control cells induced short-latency disease with splenomegaly (Fig. 1K). Loss of KDM4A significantly prolonged overall survival (OS) of mice with only one mouse succumbing to leukemia over the follow-up period by either *KDM4A*#1 KD or *KDM4A*#2 KD (Fig. 1J–K and S1F–S1H). Together, these data demonstrate a specific and essential role for KDM4A in AML cell survival.

### Targeting KDM4A’s demethylase activity inhibits AML cell proliferation

**Fig. 2:**
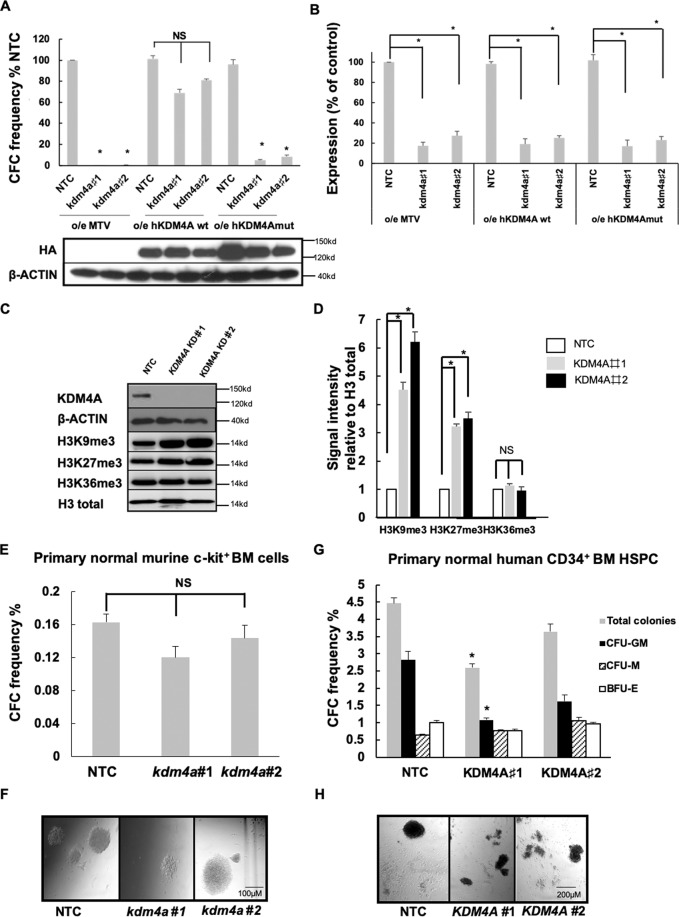
Targeting KDM4A’s demethylase activity inhibits AML cell proliferation. **A** CFC frequencies for control and *kdm4a* KD cells from the indicated murine MLL-AF9 cells overexpressing empty vector (MTV) or wild-type human HA-tagged-KDM4A or an enzymatically inactive mutant of human HA-tagged-KDM4A (KDM4Amut H188A/E190A) (*n* = 3); *p < 0.001, ^NS^*p* > 0.05. Representative immunoblot below bar plot shows the overexpression of wild type (wt) and mutant (mut) human HA-tagged KDM4A in correlated MLL-AF9 cells labeled, detected by HA antibody. **B** Bar chart showing mean ± s.e.m. an expression of *kdm4a* by QPCR in *kdm4a* KD cells from (**A**) relative to NTC in murine MLL-AF9 leukemic cells (*n* = 3); *p < 0.01. **C** Representative immunoblots with indicated antibodies showing expression of indicated proteins in THP1 cells 72 h following initiation of *KDM4A* KD (*n* = 3). **D** Immunoblot quantification of signal intensity relative to H3 total from **C**. **E**–**H** The indicated primary human and murine AML cells were transduced with lentiviruses targeting *KDM4A* or *kdm4a* for KD, or an NTC. All bar charts show mean ± s.e.m. CFC frequencies of (**E**) primary normal murine c-kit^+^ BM cells for NTC and *kdm4a* KD (*n* = 3) or (**G**) primary normal human CD34^+^ HSPC cells for NTC and *KDM4A* KD cells (*n* = 3); *p < 0.01. **F** and **H** are representative images from (**E**) and (**G**), Scale bar represents 100 µm and 200 µm, respectively.

Functional rescue experiments determined that the demethylase activity of KDM4A is required for AML. Forced-expression of wild-type human KDM4A rescued the clonogenic activity of AML cells transduced with *kdm4a* KD virus (Fig. 2A and B). This rescue phenotype was not observed by a catalytically inactive mutant of KDM4A (KDM4A^H188A/E190A^)^19,20^ (Fig. 2A and B) in murine MLL-AF9 cells. Next, we assessed KDM4A substrates H3K9me3 and H3K36me3 in *KDM4A* KD THP1 cells. There was an increase in H3K9me3 shown by immunoblotting (Fig. 2C and D) and immunofluorescent staining (Figs. S2A–S2B). No significant changes in H3K36me3 were observed (Fig. 2C–D; S2A–S2B), suggesting H3K9me3 is the primary target of KDM4A in THP1 cells. In addition, there was a marked elevation of H3K27me3 in *KDM4A* KD THP1 cells (Fig. 2C–D; S2A–S2B) and two further *KDM4A* KD human MLLr-AML cell lines (Figs. S2C–S2D).

We next investigated whether KDM4A is dispensable for normal hematopoiesis. Our data showed no significant loss of colonies in *kdm4a* KD normal murine BM c-kit^+^ cells in CFC assays (Fig. 2E and F; S2E). Similarly, reduced levels of *KDM4A* in human CD34^+^ HSPCs from healthy donors are tolerated (Fig. 2G and H; S2F) with fewer colonies due to a reduction of CFU-GM in *KDM4A* KD #1 cells. Although a KDM4A specific inhibitor is lacking, there are several pan-KDM4 inhibitors available including IOX1^21^ and IOX derivatives (IOX1^dev^)^22,23^. These displayed significant inhibitions of cell proliferation in THP1 cells and primary AML patient blasts, inducing differentiation and apoptosis (Figs. S3A–S3G) with minimum effect on normal human CD34^+^ BM HSPCs (Fig. S3E). These phenotypes were accompanied by an increased level of H3K9me3 and H3K27me3 (Fig. S3H), suggesting the anti-leukemic effect was due to KDM4A inhibition.

### PAF1 identified as a KDM4A co-regulator is required for human AML cell survival

**Fig. 3:**
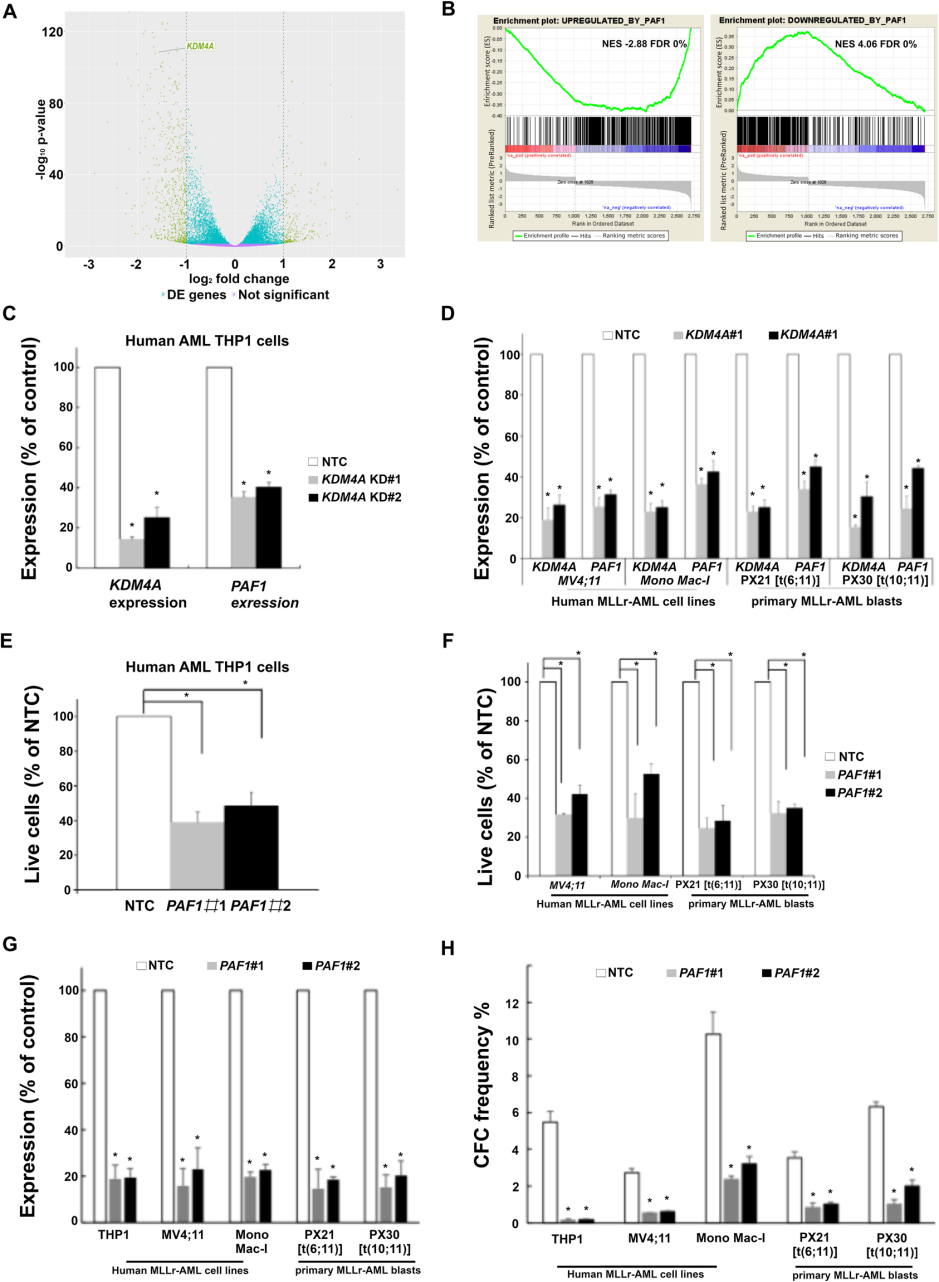
*PAF1* identified as a cofactor of KDM4A in MLLr-AML. **A** Volcano plot showing global changes in gene expression following the loss of *KDM4A* compared to NTC control THP1 cells as identified by RNAseq. The absolute number of upregulated or downregulated genes that are bound by KDM4A is indicated at the top right and left side of the plot, respectively (KDM4A bound: FDR ≤ 0.01, gene expression: log_2_ FC ≥0.5 or ≤−0.5; adjusted (adj. *p*) *p* ≤ 0.05). **B** GSEA show overlapping transcriptional consequences following the loss of *KDM4A* or *PAF1* in THP1 cells. Specifically, genes repressed (left panel) or activated (right panel) are upregulated and downregulated, respectively, following the loss of KDM4A^26^. **C**–**H** THP1 cells and other indicated human AML cells were transduced with lentiviruses targeting *KDM4A* or *PAF1* for KD, or an NTC. All bar charts show mean ± s.e.m. **C** Bar chart showing relative expression of *KDM4A and PAF1* by QPCR in comparison with NTC control cells following *KDM4A* KD using two different shRNA constructs ♯1 and ♯2 in THP1 cells (*n* = 3) (**D**) and the other indicated human AML cells and primary AML cells include PX21, containing t(6;11)(MLL-AF6) chromosomal translocation and PX30 t(10;11)(MLL-AF10) (PX number is the Paul O’Gorman Leukaemia Research Centre Biobank sample identifier) (*n* = 3) (**F**); *p < 0.01. **E**–**F** Percentage of live cell counts in comparison with NTC control (**E**) in THP1 cells 4 days following lentiviral infection (*n* = 3) and (**F**) in the indicated human MLLr-AML cell lines and AML primary cells following *KDM4A* KD in comparison with NTC control cells (*n* = 3) (**I**) *p < 0.01. **G** Bar chart showing relative expression of *PAF1* by QPCR in comparison with NTC control cells following *PAF1* KD using two different shRNA constructs ♯1 and ♯2 in (**E** and **F**). **H** Bar chart showing the loss of CFC frequencies of indicated human AML cell lines and primary patient samples (*n* = 3) following lentivirus infection, puromycin selection, and initiation of *PAF1* KD; *p < 0.001.

To determine the impact of KDM4A on global gene expression, we compared the transcriptome of *KDM4A* KD THP1 cells with NTC control by RNA-seq. 3375 differentially expressed (DE) genes were significantly deregulated following depletion of *KDM4A* (Log_2_ fold change (FC) ≥0.5 or ≤−0.5; adj. *p* ≤ 0.05; Fig. 3A; Supplemental file). 67% (2274 out of 3375) were direct targets of KDM4A; ChIP-seq revealed that KDM4A bound at their TSS (Supplemental file). 61% (1387 out of 2274) of putative KDM4A direct target genes were downregulated associated with the enrichment of transcriptional repressive marks at H3K9me3^24^ and H3K27me3^25^ (Fig. 3A).

To provide insights into the survival pathways regulated by KDM4A, we performed gene-set enrichment analysis (GSEA) and revealed significant enrichment of genes regulated by PAFc^26–28^ (Fig. 3B). This is consistent with downregulation of *PAF1*, following *KDM4A* KD at the transcript (Fig. 3C and D) and protein (Fig. S4A) level in human MLLr-AML cell lines, and primary patient blasts. *PAF1* KD phenocopied *KDM4A* KD in MLLr-AML cells, inducing significant apoptosis (Fig. 3E–G; S4B) and loss of CFU potential (Fig. 3H). Together, these data suggest loss of KDM4A impairs PAF1 function to maintain leukemic cell survival, supporting PAF1 as an important cofactor of KDM4A in human AML.

### KDM4A-PAF1 maintains appropriate expression of the MLLr-fusion oncogenic program in MLLr-AML

**Fig. 4:**
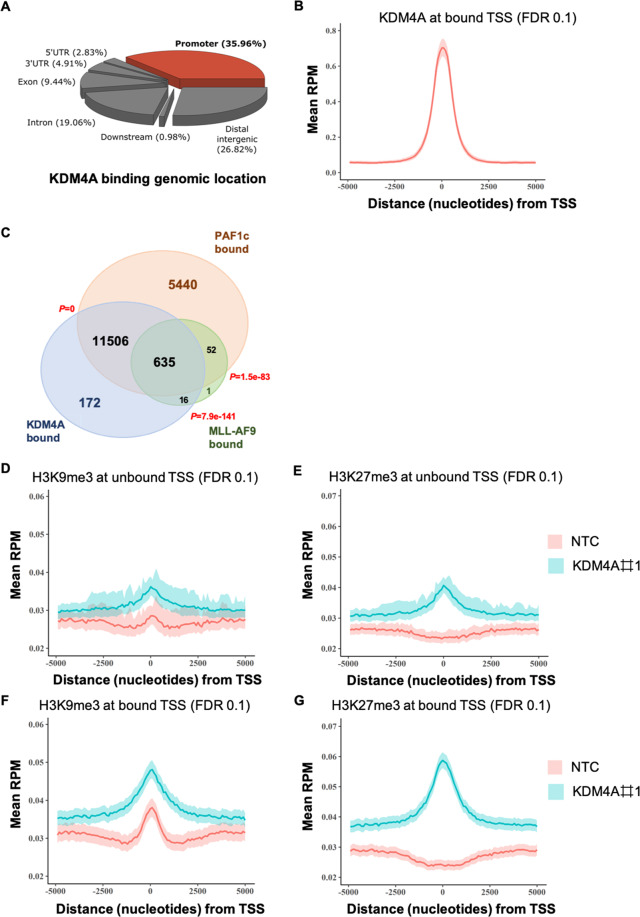
KDM4A-PAF1 co-regulates essential MLLr-fusion oncogenic transcriptional program. **A** Feature distribution of KDM4A ChIP-seq peaks in the THP1 cell genome. **B** Metagene plots showing a distinct peak in KDM4A normalized ChIP-seq signal in reads per million mapped reads (RPM) at transcription starting sites (TSS) in WT THP1 cells. **C** Venn diagram showing the overlap between binding sites of KDM4A, PAF1c^26^, and MLL-AF9^29^ in THP1 cells as determined by ChIP-seq; *p* by hypergeometric test. **D**–**G** Metagene plots showing enrichment of H3K9me3 (**F**) and H3K27me3 (**G**) at KDM4A bound TSS compared to unbound TSS (**D** and **E**) following *KDM4A* KD by ChIP-seq.

**Fig. 5:**
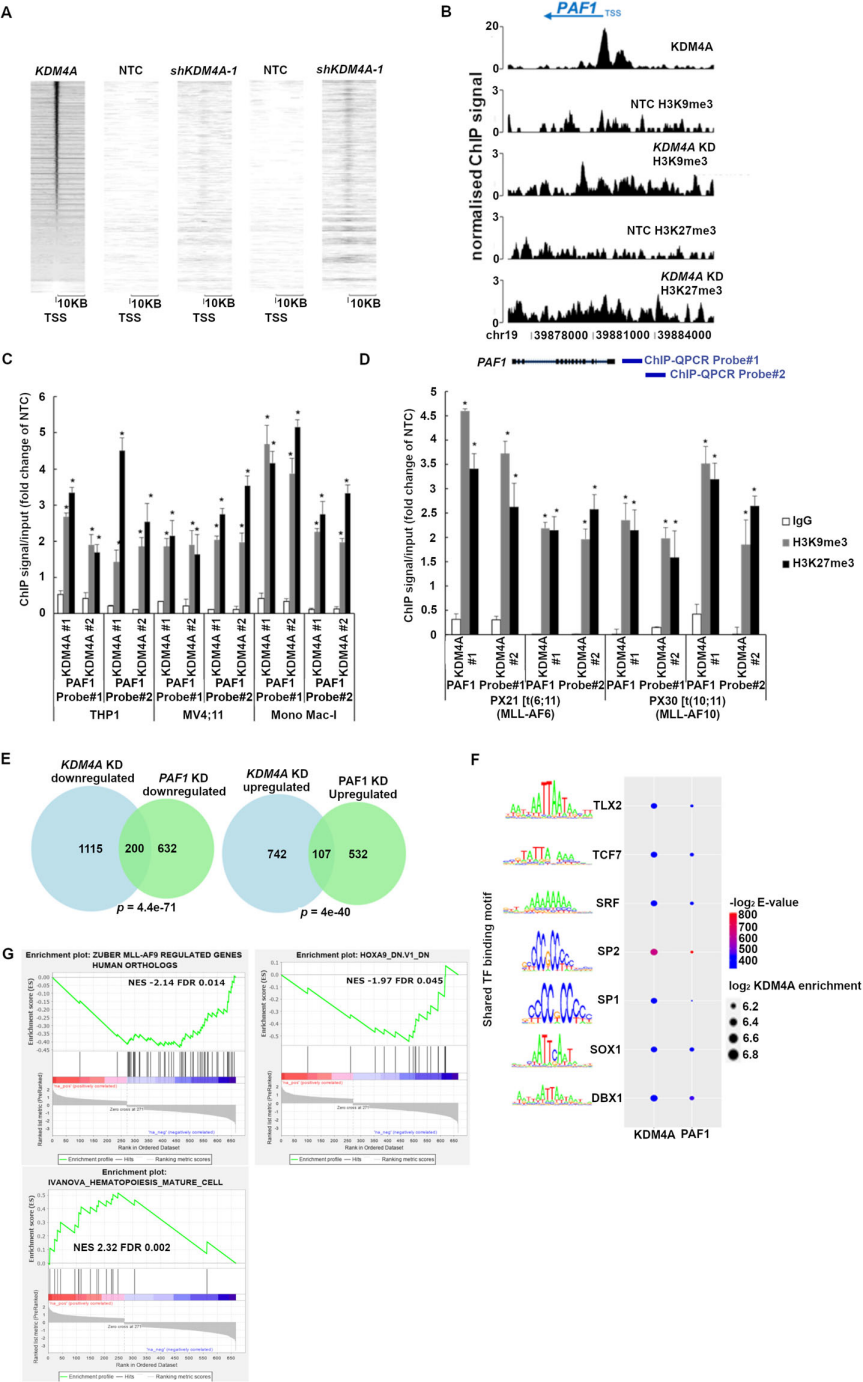
KDM4A-PAF1 maintains appropriate expression of the MLLr-fusion oncogenic program in MLLr-AML. **A** Heatmap showing normalized ChIP-seq signal of H3K9me3 and H3K27me3 at TSS across all genes in *KDM4A* KD and NTC THP1 cells ordered by KDM4A enrichment. **B** Genomic snapshot demonstrates KDM4A occupancy at the PAF1 promoter region and enrichment of H3K9me3 and H3K27me3 signal throughout the PAF1 gene body and promoter upon *KDM4A* KD in comparison with NTC control in THP1 cells. Blue bars show the two individual probes used for ChIP-QPCR in **C** and **D**. **C**–**D** H3K9me3 and H3K27me3 ChIP signal/input (fold change of NTC) in the indicated human MLLr-AML cell lines (**C**) and indicated primary AML samples including PX21 (MLL-AF6) and PX30 (MLL-FA10) (**D**) as determined by ChIP-QPCR following depletion of *KDM4A*; *p < 0.001. **E** Venn diagrams showing the overlap between directly bound downregulated and upregulated targets of KDM4A and the PAF1c^24^ in THP1 cells following knockdown of *KDM4A* and *PAF1* as determined by ChIPseq; *p* by hypergeometric test. **F** Motif significance and KDM4A log_2_ enrichment at KDM4A or PAF1 regulated promoters (FDR ≤ 0.01, DE ≤ −0.5, or DE ≥ 0.5). Color represents motif significance within KDM4A and PAF1 regulated promoters. Size denotes the average log_2_ enrichment of KDM4A within each group of promoters that possess the respective transcription factor (TF) binding motif. Top five motifs detected in KDM4A or PAF1 regulated promoters sorted by statistical significance (*E*-value). **G** GSEA results showing significant overlap of *KDM4A* KD transcriptional consequences with downregulation of MLL-AF9 and HOXA9 targets and upregulation of a mature hematopoiesis program in THP1 cells, *q < 5%.

Our ChIP-seq data reveal substantial overlap amongst PAFc^26^, MLL-AF9^29^, and KDM4A binding sites (Fig. 4A–C). Specifically, KDM4A bound the PAF1 promoter region (Supplemental file), suggesting a direct regulatory mechanism. There is no enrichment of either histone methylation mark at non-KDM4A binding genomic loci (Fig. 4D and E), indicative of a human KDM4A-specific epigenomic profile. In marked contrast, there is a global gain of both H3K9me3 and H3K27me3 upon *KDM4A* KD in THP1 cells at KDM4A binding sites (Fig. 4F and G; 5A), including the genomic loci of PAF1 and its targets (Fig. 5B). These were validated by ChIP-QPCR in cell lines and primary patient blasts (Fig. 5C and D).

Furthermore, genes with significant expression changes following KDM4A silencing were also enriched in direct PAF1 target genes^26^ (Fig. 5E), suggesting a transcriptional network co-regulated by both KDM4A and PAF1. This notion is supported by the fact that KDM4A bound promoters share almost identical enrichment of transcription factor (TF) binding motifs as the ones bound by PAF1, including homeobox (HOX) TFs, such as TLX2 and DBX (Fig. 5F). Further GSEA analysis on the overlapped DE genes between *KDM4A* KD and *PAF1* KD revealed a significant downregulation of MLLr fusion target genes^30^, as well as HOX family target genes^31^ including notably the pro-survival gene, *BCL2*, and marked upregulation of a mature hematopoiesis program^32^ (Fig. 5G) and pro-apoptotic gene, *BCL2L11* (*BIM*). Although the expression of *HOXA9* itself was not affected by either KD, our data suggest KDM4A and PAF1 co-regulate their downstream targets in a parallel manner.

### A core 9-gene signature downstream of *KDM4A* strongly associated with LSC activity and clinical outcome

**Fig. 6:**
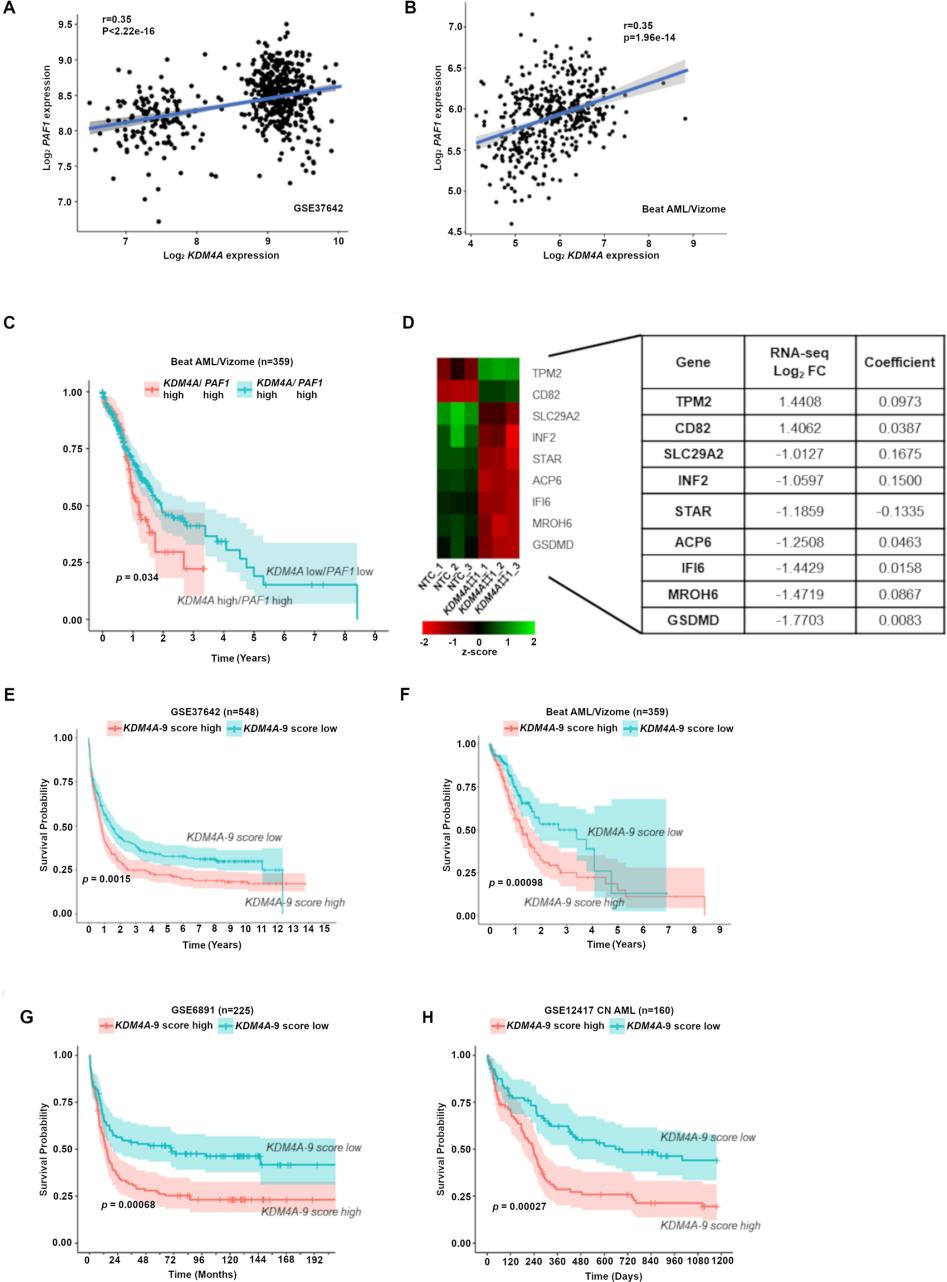
A core 9-gene signature downstream of *KDM4A* strongly associated with clinical outcome. **A**–**B** Scatterplot showing the correlation between expression of *KDM4A* versus *PAF1* in primary AML patient samples (GSE37642) (**A**) and (Beat AML/Vizome), R by Pearson correlation, *p* < 0.05 (**B**). **C** Kaplan-Meier survival analysis conducted in Beat AML dataset. Patients with both *KDM4A*^high^ and *PAF1*^high^ expression have inferior overall survival. Patients dichotomized into high and low groups for *KDM4A* or *PAF1* based on whether expression was above the median for each gene; *p* by log-rank test. **D** Heatmap showing gene expression of the *KDM4A-9* gene signature genes with the table of their respective regression coefficients and log_2_ FC as determined by RNA-seq in *KDM4A* KD THP1 cells. **E**–**H** Kaplan-Meier survival analysis conducted in the large AML datasets (GSE37642) (**E**), (Beat AML/Vizome) (**F**), (GSE6891) (**G**), and (GSE12417) (**H**) showing that the *KDM4A-9* score can predict survival across AML patients of varying subtypes. Patients were dichotomized into high and low groups based on whether they possessed a score above or below the median signature score; *p* by log-rank test.

Supporting the collaborative role of KDM4A-PAF1 in AML, *KDM4A* expression is highly associated with *PAF1* expression in patient datasets (Fig. 6A and B); *KDM4A-PAF1* expression can identify patients with inferior OS (Fig. 6C). *KDM4A* KD induced a significant reduction of cell proliferation in human AML cell lines representative of different subtypes (Fig. S5A), coupled with an increase in apoptosis and loss of CFC potential (Figs. S5B and S5C). This evidence suggests that KDM4A is required across AML.

**Fig. 7:**
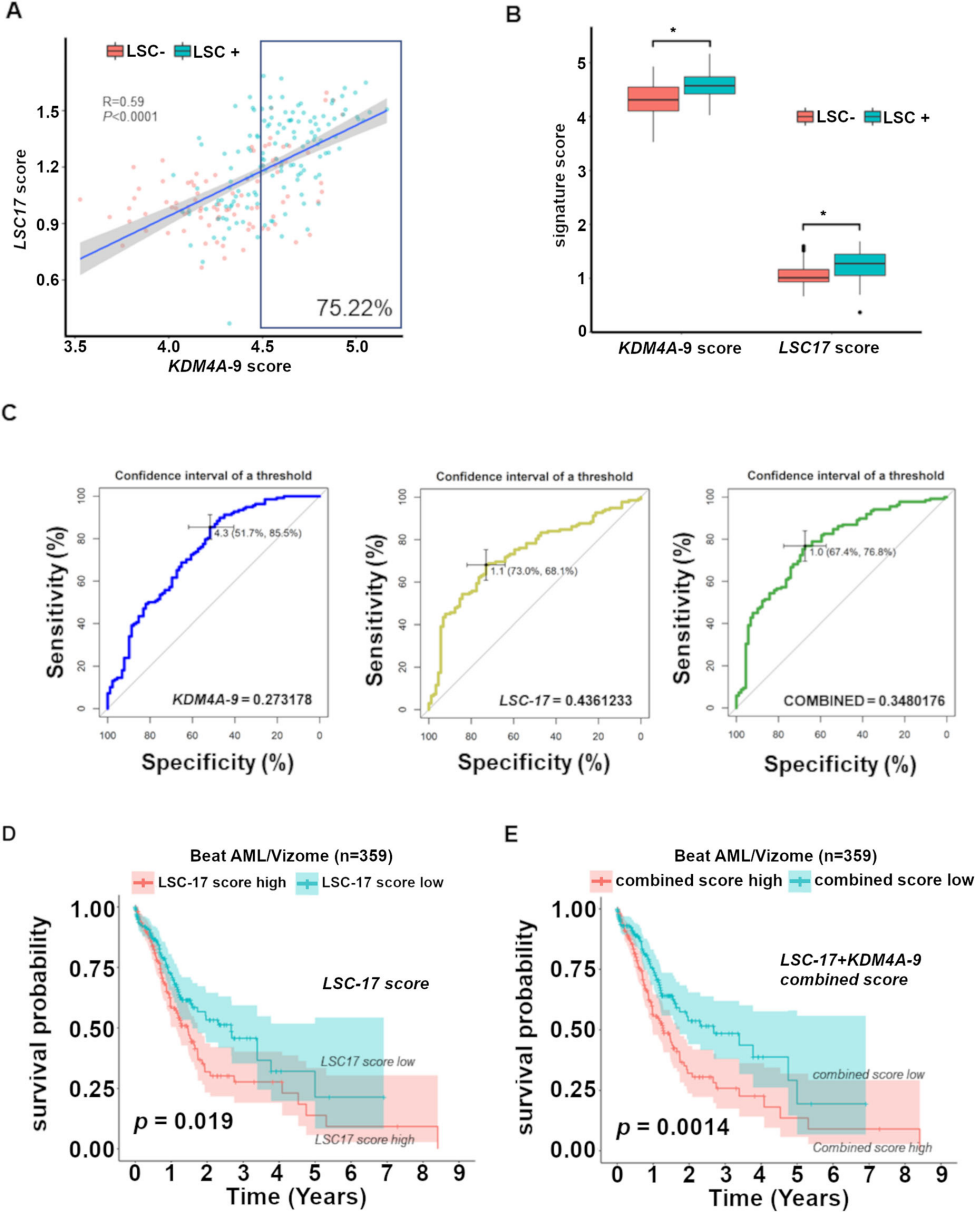
*KDM4A-9* enriched with LSC activity, is a poor prognosis marker for AML. **A** Scatterplot showing a moderate correlation between the *KDM4A-9* score and *LSC17* score in primary AML patient samples (GSE76008). LSC enriched (LSC+, *n* = 138) cell fractions from 78 patient samples are colored blue whilst those that lack LSC enrichment (LSC−, *n* = 89) are colored red. Over 75% of *KDM4A-9* high score (above median value) fractions are LSC+. Pearson correlation used to assess correlation. Significance determined by *t*-test. **B** Box plot showing *KDM4A-9* or *LSC17* signature scores in two comparative groups: LSC+ and LSC− from (**A**); unpaired *t*-test, *p < 0.0001. **C** ROC curves of *KDM4A-9* (blue), *LSC17* (yellow), and *KDM4A-9/LSC17* (green) show the diagnostic capability of each signature to predict LSC enrichment in AML samples. The black bars in each plot are the 95% confidence intervals for the optimal cut-off. The Youden index was used to determine the optimal cut-off for each signature. **D**–**E** Patients in the Beat AML/Vizome dataset were dichotomized into high and low groups based on whether they possessed a score above or below the median signature score. Kaplan-Meier survival analysis conducted showing that the combined *KDM4A-9*/*LSC17* score (**E**) is effective in the prediction of AML patient survival over the *LSC17* score alone (**D**).

These data led to our hypothesis that a core gene expression signature (GES) downstream of the *KDM4A-PAF1* regulatory axis, is associated with AML patient outcomes compared with the known LSC score, *LSC17*^17^. For this, we utilized LASSO regression analysis^16,17,33^ on KDM4A regulated genes (*KDM4A* KD Log_2_FC ≥1 or ≤−1; adj. *p* ≤ 0.05; Supplemental file) and defined a *KDM4A-9* score (Fig. 6D). High *KDM4A-9* was highly associated with poor OS in AML cohorts (Fig. 6E–H) independent of age, cytogenetic risk score, and frequent mutation status. The robust prognostic value of *KDM4A-9* indicates that the score may be related to the important biological activities of AML-LSCs. Indeed, *KDM4A-*9 correlates with the *LSC17* score of AML samples and over 75% of *KDM4A-9* high score fractions are LSC+ (Fig. 7A), therefore *KDM4A-9* is a strong predictive indicator of AML-LSC activity (Fig. 7B). Interestingly, there is no overlap between these two gene signatures. A combined signature score (*KDM4A-9/LSC17*) achieves an optimal balance between specificity and sensitivity (Fig. 7C) overcoming the limitations of either score alone with an improved ability to predict survival over *LSC17* (Fig. 7D and E).

**Fig. 8:**
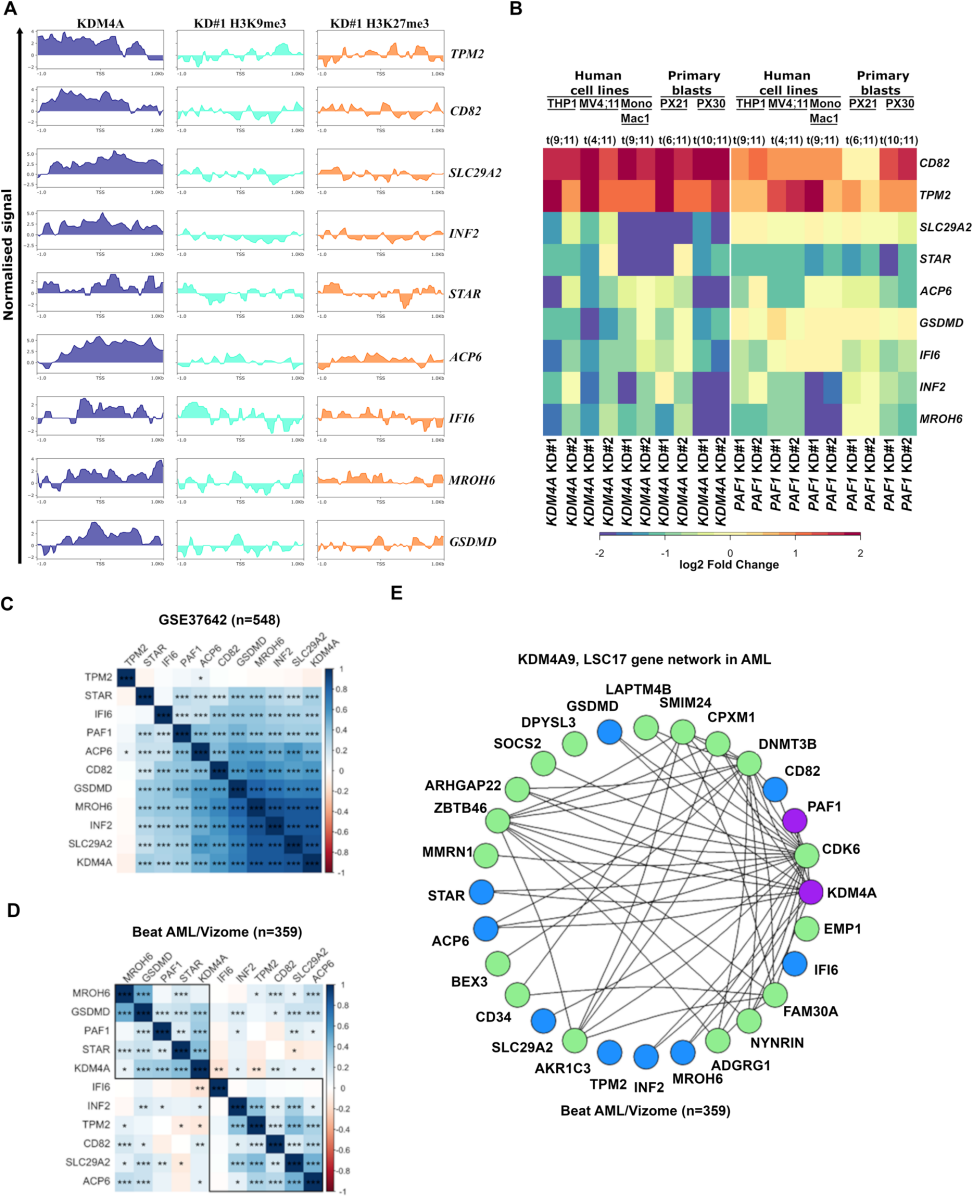
KDM4A-mediated epigenomic network required for AML cell self-renewal and survival. **A** Heatmap showing relative expression of *KDM4A-9* signature genes as determined by QPCR in the indicated human MLLr-AML cell lines and AML primary cells following *KDM4A* KD or *PAF1* KD in comparison with NTC control cells (*n* = 3). **B** Input normalized ChIP-seq coverage tracks showing KDM4A ChIP signal in WT THP1 cells and H3K9me3/H3K27me3 ChIP signal normalized to NTC in *KDM4A* KD THP1 cells at *KDM4A-9* signature genomic loci (+/−1 kb TSS). The normalized signal shown is the log_2_ ratio of read counts compared against input control. **C**–**D** Correlation matrices showing the Pearson correlation coefficients for *KDM4A*, *KDM4A*-9 genes, and *PAF1* gene expression in GSE37642 (**C**) and Beat AML/Vizome (**D**) AML datasets. Significance determined by *t*-test; *p < 0.05, **p < 0.01, ***p < 0.001, ****p < 0.0001. **E** *KDM4A*, *PAF1*, *KDM4A-9* (in blue), and *LSC17* (in green) gene network showing the topological overlap between genes as detected from 262 AML samples (Beat AML) (the corresponding topological overlap matrix (TOM) ≥ 0.05 between nodes. Genes with high topological overlap matrix (TOM) measure (TOM ≥ 0.05) have related functions).

KDM4A binds at the promoter regions of *KDM4A*-9 genes, whereupon H3K9me3 and H3K27me3 are enriched after *KDM4A* KD (Fig. 8A; Supplemental file), suggesting direct regulation. In addition to the QPCR validation in human AML cell lines and primary AML blasts following *KDM4A* KD or *PAF1* KD (Fig. 8B), we observed that the majority of *KDM4A-9* genes show correlation with *KDM4A and PAF1* expression in patient AML cohorts (Fig. 8C and D). Furthermore, weighted gene correlation network analysis (WCGNA)^34^ demonstrated a strong relationship between *KDM4A*, *PAF1*, and the *KDM4A-9* and *LSC17* GESs across AML. This network possessed high topological overlap (topological overlap matrix (TOM) ≥0.05)) with KDM4A as a highly connected node (Fig. 8E) suggesting that KDM4A-PAF1 regulates the *KDM4A-9*/*LSC17* network in AML.

### KDM4A has a distinct function to another KDM4 family member, KDM4C in AML

Previously, Cheung et al. showed that KDM4C is required for MLLr-AML cell survival^8^, indicating an overlapping role of KDM4A and KDM4C in AML. However, forced-expression of wild-type human KDM4C failed to rescue the clonogenic activity of murine MLL-AF9 AML cells transduced with *kdm4a* KD virus (Figs. S6A and S6B), suggesting KDM4A has a distinct role from that of KDM4C. This is in line with previously reported data showing no increase of global H3K27me3 level upon pharmacological inhibition of KDM4C in MLLr-AML cells^8^. Consistently, at the molecular level, *KDM4A* KD led to transcriptional changes distinct from *KDM4C* KD via GSEA comparison (Fig. S6C), further supporting a unique role for KDM4A compared to KDM4C in human AML. In particular, *KDM4A* KD has no significant impact on gene expression of two established targets of KDM4C, *HOXA9*, and *MEIS1* in human MLLr-AML cells. These results are also validated by Q-PCR using shRNAs targeting *HOXA9* as control (Fig. S6D). More importantly, *kdm4c* KD had no impact on *PAF1* expression, nor its associated genes targeted by KDM4A including *KDM4A-9* and *LSC17* GESs (Fig. S6E). Together, these data demonstrate an essential role of KDM4A in human AML.

## Discussion

Previous reports indicate that the KDM4 family is required for normal hematopoiesis^9,35^, whilst loss of individual members is tolerated in normal cells^35^ highlighting the importance of identifying KDM4 family members that are essential for the survival of AML cells. Our data demonstrate KDM4A is unique; it is selectively required for AML cell survival, with no negative effect on normal hematopoiesis offering a therapeutic window. Lack of tractable enzymatic activities limits the potential of PAF1 or other subunits of the PAFc as therapeutic targets in cancer. Herein, we identify a novel *KDM4A-PAF1* signaling axis co-regulating oncogenic transcriptional networks in human AML, providing a way to eliminate leukemic cells with broad therapeutic applications. Our study provides a strong rationale for the further development of KDM4A inhibitors, presenting a promising strategy for novel epigenetic-based therapy in AML.

*KDM4A-9* shows strong therapeutic implications comparable with *LSC17*^17^. A high *KDM4A-9* score may reflect an important biological property of KDM4A in leukemogenesis. The function of the *KDM4A-9* genes in leukemogenesis is unknown; except Tetraspanin (CD82)^36,37^ which plays an important role in AML. Corroborating recent findings^5,6^, our data suggest KDM4A regulates H3K9me3 to direct leukemogenesis. H3K9me3 has emerged as a key player in repressing lineage-inappropriate genes, impeding the reprogramming of cell identity during development and cell fate determination^24,38^. It would be interesting to determine further the clinical diagnostic relevance of H3K9me3 in relation to KDM4A in AML patients.

## Supplementary information

Supplemental Material

Supplemental gene expression excel file
