# Supplementary material for: A KDM4A-PAF1-mediated epigenomic network is essential for acute myeloid leukemia cell self-renewal and survival
Source: Cell Death Dis. 2021 Jun 3;12(6):573. doi: 10.1038/s41419-021-03738-0 (PMC8175737; doi:10.1038/s41419-021-03738-0)
Supplement: Supplementary file 1 — Supplemental Material [file 41419_2021_3738_MOESM1_ESM.docx]

**Supplemental Data**

**A KDM4A-PAF1-mediated epigenomic network is essential for acute myeloid leukemia cell self-renewal and survival**

Matthew E Massett, Laura Monaghan, Shaun Patterson, Niamh Mannion, Roderick P Bunschoten, Alex Hoose, Sandra Marmiroli, Heather G Jørgensen, Robert MJ Liskamp, David Vetrie, Alison M Michie, Xu Huang

**Contents**

Supplemental Table 1. List of lentiviral constructs for knockdown experiments from Sigma

Supplemental Table 2. List of antibodies

Supplemental Table 3. List of AML patient datasets used in this study

Supplemental Figures

Figure S1.

Figure S2.

Figure S3.

Figure S4.

Figure S5.

Figure S6.

Supplemental excel file- gene lists in RNA-seq and ChIP-seq analysis (in a separate file)

| **shRNA clone** | **Sigma TRC number** |
| --- | --- |
| NTC | SHC002 |
| *KDM4A*♯1 | TRCN0000234911 |
| *KDM4A*♯2 | TRCN0000234914 |
| *KDM4A*♯3 | TRCN0000234912 |
| *KDM4A*♯4 | TRCN0000234913 |
| *KDM4A*♯5 | TRCN0000234910 |
| *PAF1*♯1 | TRCN0000342666 |
| *PAF1*♯2 | TRCN0000342733 |
| *kdm4a*♯1 | TRCN0000103526 |
| *kdm4a*♯2 | TRCN0000103528 |
| *MLL*♯1 | TRCN0000005955 |
| *MLL*♯2 | TRCN0000005956 |
| *MEN*♯1 | TRCN0000040140 |
| *MEN*♯2 | TRCN0000040141 |

**Supplemental Table 1. List of lentiviral constructs for knockdown experiments from Sigma**

| **Antibody** | **Immunoblot and IF** | **FACS** | **ChIP-seq** |
| --- | --- | --- | --- |
| CD13-PE | N/A | eBioscience  #12-0138-42 | N/A |
| CD86-AF488 | N/A | eBioscience  #53-0869-42 | N/A |
| H3K9me3 | Cell Signalling #13969s | N/A | Diagenode #C15410056 |
| H3K27me3 | Cell Signalling #9733 | N/A | Diagenode #154100195 |
| H3K36me3 | Cell Signalling #9763s | N/A | Diagenode #15410058 |
| KDM4A | Cell Signalling #3393s | N/A | Bethyl #A300-860A |
| PAF1 | Santa Cruz #514491 | N/A | N/A |
| β-ACTIN | Cell Signalling #D6A8 | N/A | N/A |
| HA-tag | Cell Signalling #2367 | N/A | N/A |
| H3 total | Cell Signalling #14269 | N/A | N/A |

**Supplemental Table 2. List of antibodies**

| **Dataset** | **Data type** | **Description** | **Number of signature validation samples** |
| --- | --- | --- | --- |
| GSE76008(ref. 1) | Microarray | CD34/CD38 sorted AML samples with characterised LSC activity based on xenotransplantation assays. | N/A |
| GSE6891(ref. 2) | Microarray | De novo AML | 225 |
| GSE12417(ref. 3) | Microarray | Cytogenetic Normal Adult AML | 160 |
| GSE37642(ref. 4) | RNA-seq | AML patients at diagnosis or after treatment. | 539 |
| Beat AML/Vizome(ref. 5) | RNA-seq | AML patients at diagnosis or after treatment. | 359 |
| E-MTAB-3322 | RNA-seq | *Kdm4c* KD in HSPC transformed with the MLL-AF9 fusion oncoprotein. | N/A |
| [GSE62171](https://www.ncbi.nlm.nih.gov/gds/?term=GSE62171%5bAccession%5d) | ChIP-seq / RNA-seq | PAF1 KD in THP-1 cells | N/A |
| GSE79899 | ChIP-seq | MLL ChIP-seq in THP-1 cells | N/A |

**Supplemental Table 3. List of AML patient datasets used in this study**

**Supplemental Figures**

**
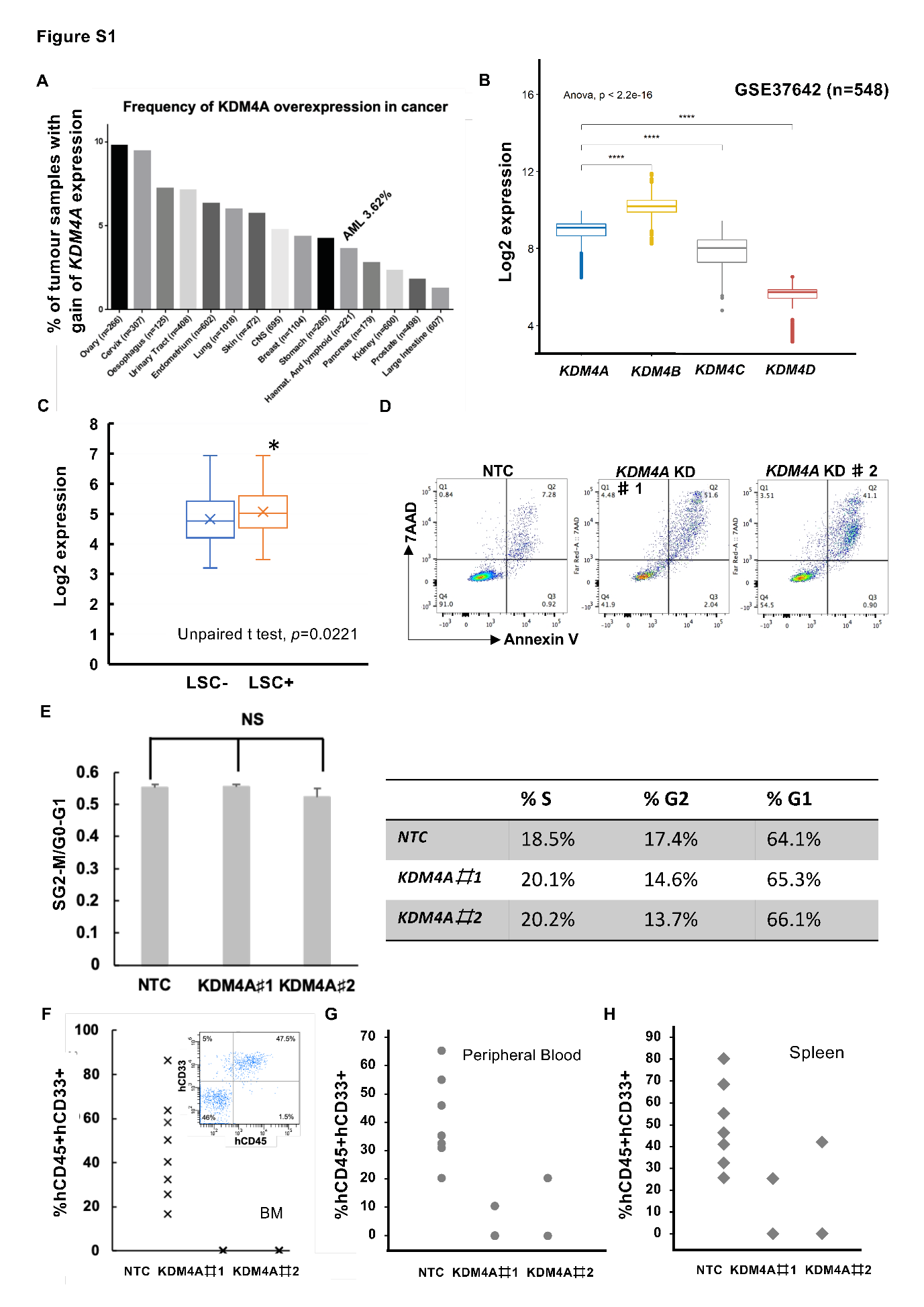
**

**Figure S1. *KDM4A* KD impedes cell proliferation and induces apoptosis in human AML cells.**

All bar charts show mean ± s.e.m**.** (A) Bar plot showing frequency of *KDM4A* overexpression in various cancers. Data obtained from COSMIC (19). Overexpression defined by samples with a z-score ≥ 2. (B) Box plot of KDM4 family gene expression in AML patients in GSE37642; **p*<0.05, ***p*<0.01, ****p*<0.001, *****p*<0.0001. (C) The 227 samples from 78 AML patients in the GSE76008 dataset are divided into LSC positive (LSC+, n=138) and negative (LSC-, n=89) groups, box plot showing *KDM4A* gene expression in two comparative groups; unpaired *t*-test, **p*=0.0221. (D) Representative FACS plots from Fig. 1E. (E-F) FACS analysis of THP1 cell following *KDM4A* depletion showing (E) no alteration in the cell cycle using propidium iodide DNA staining and an accompanying table showing mean % cells at each cell cycle phase. (F-H) Graph showing percentage human AML cell engraftment (hCD45+hCD33+ cells) in NSG mice BM (F) with representative flow cytometry plot showing primary human AML engraftment, peripheral blood (G) and spleen (H) from leukemic mice in Fig. 2J.


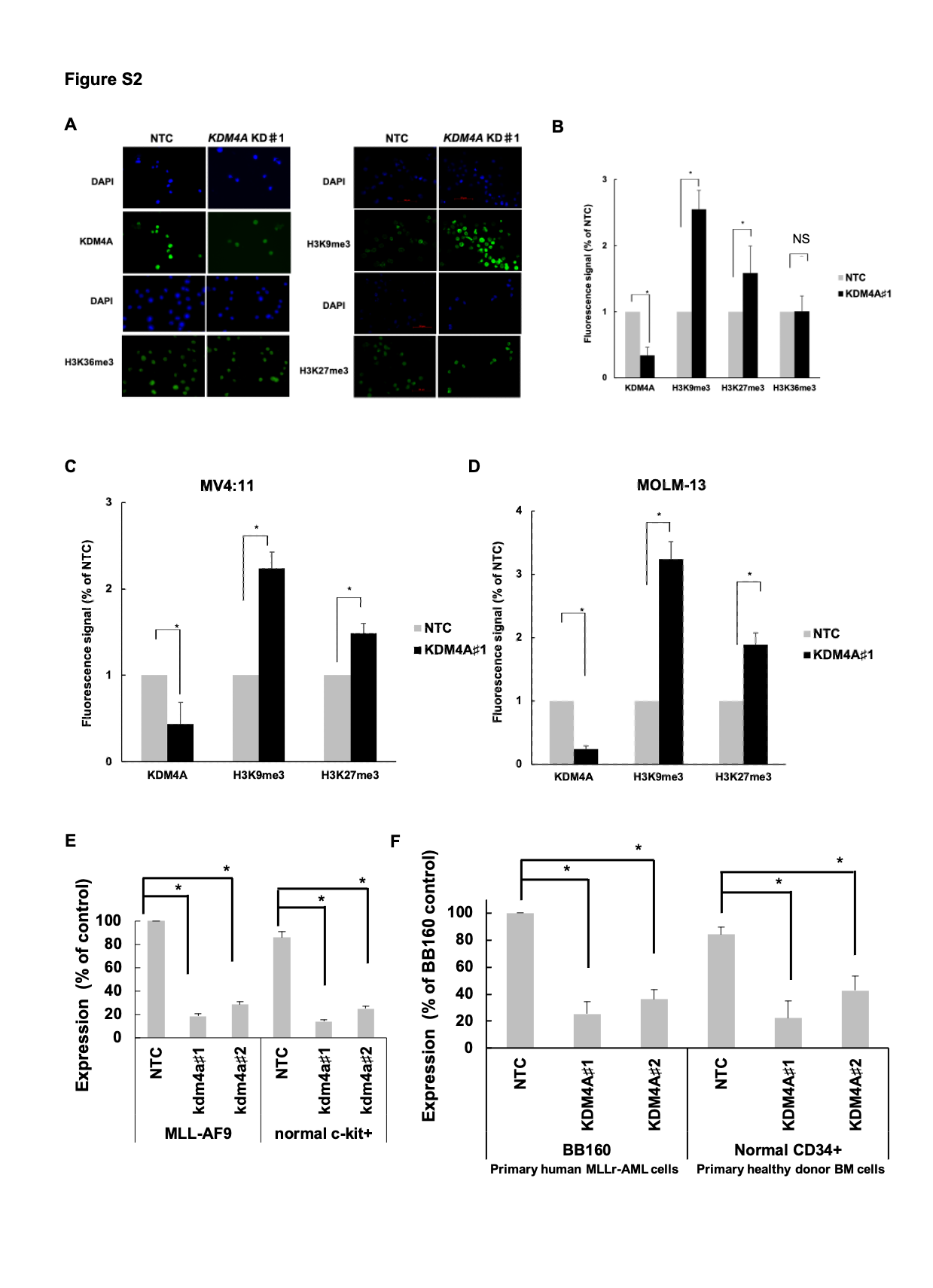
**Figure S2. KDM4A is required for the functional potential of human AML cells but less needed for normal cells.**

(A-D) Representative IF images (A) and bars showing quantified signal relative to NTC from each sample (>200 cells per condition) in human THP1 cells (B), MV4:11 (C) and MOLM-13 cells (D) following *KDM4A* KD (n=3) mean ± s.e.m. **p*< 0.001, ^NS^*p* >0.05. (E-F) Expression of *kdm4a*/*KDM4A* by QPCR in indicated KD murine cells from Fig. 2E or human cells from Fig.2G relative to NTC control cells (n=3); **p*<0.001.

**
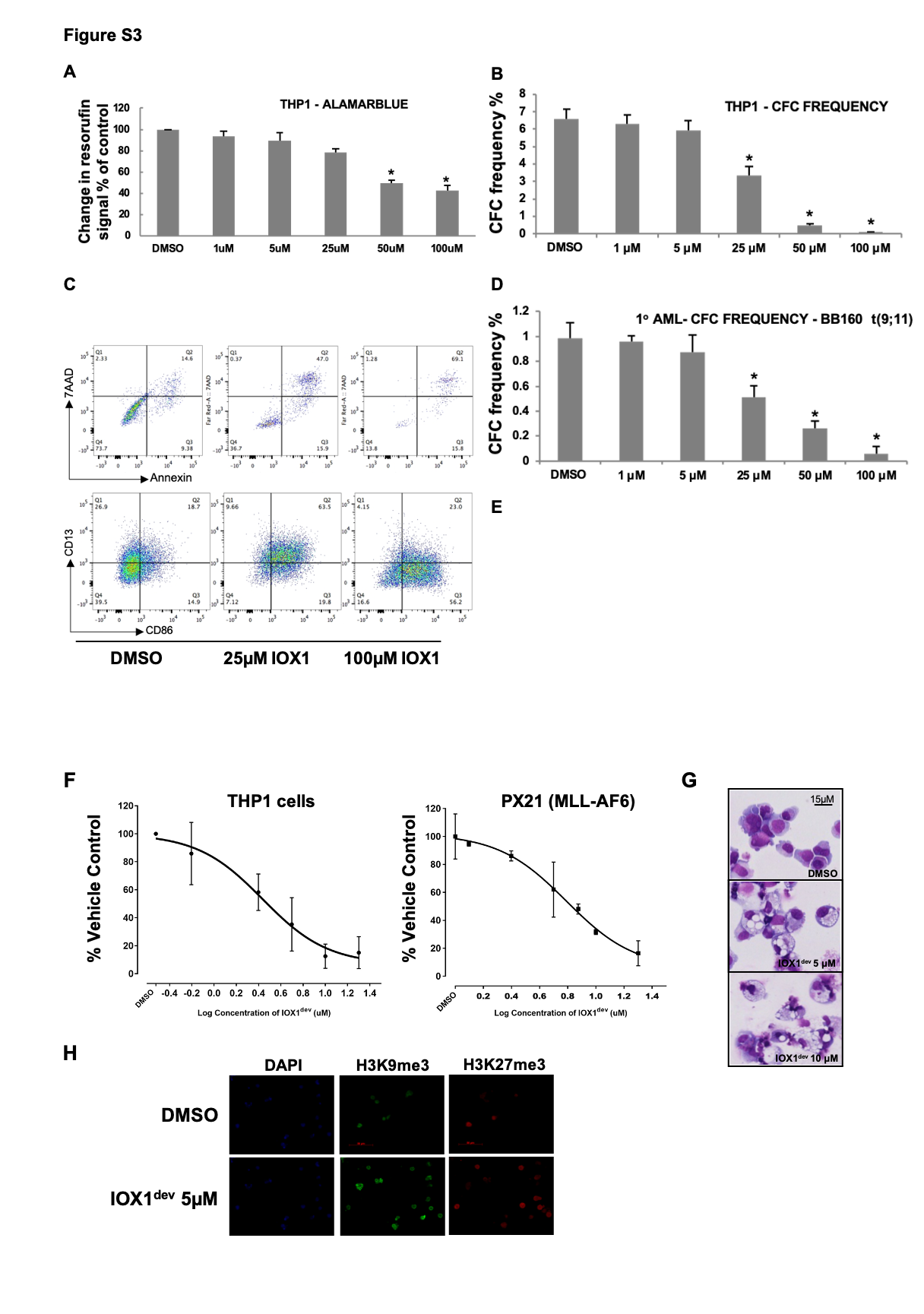
**

**Figure S3. AML cells are vulnerable to pharmacological inhibition of KDM4.**

(A) Resorufin signal after 2 days treatment with IOX1 for human THP1 AML cells relative to control cells treated with DMSO (n=3). (B) Reduction in CFC frequency following treatment with indicated concentrations of IOX1 in human THP1 AML cells relative to control cells treated with DMSO. (C) Representative FACS analysis of the percentage of apoptotic cells and markers of differentiation determined by Annexin V^+^/ 7AAD^+/-^ staining (top panel) and differentiation (lower panel) respectively after IOX1 inhibitor treatment (n=3). (D-E) Bar plots showing reduction in CFC frequencies in primary AML samples, BB160 (MLL-AF9), **p*<0.01**.** (D) and no significant effect on normal human CD34^+^ BM HSPCs (E) following treatment with indicated concentrations of IOX1 (n=3). (F) Dose-response curves determined from Resorufin cell proliferation assays using the pan-KDM4 inhibitor IOX1^dev^ in THP1 cells and primary AML cells, PX21 (MLL-AF6) showing efficacy of KDM4 inhibition in human AML. DMSO was used as a vehicle control. (G) Representative cytospin images and IF images (H) of the indicated proteins in THP1 cells from (G).


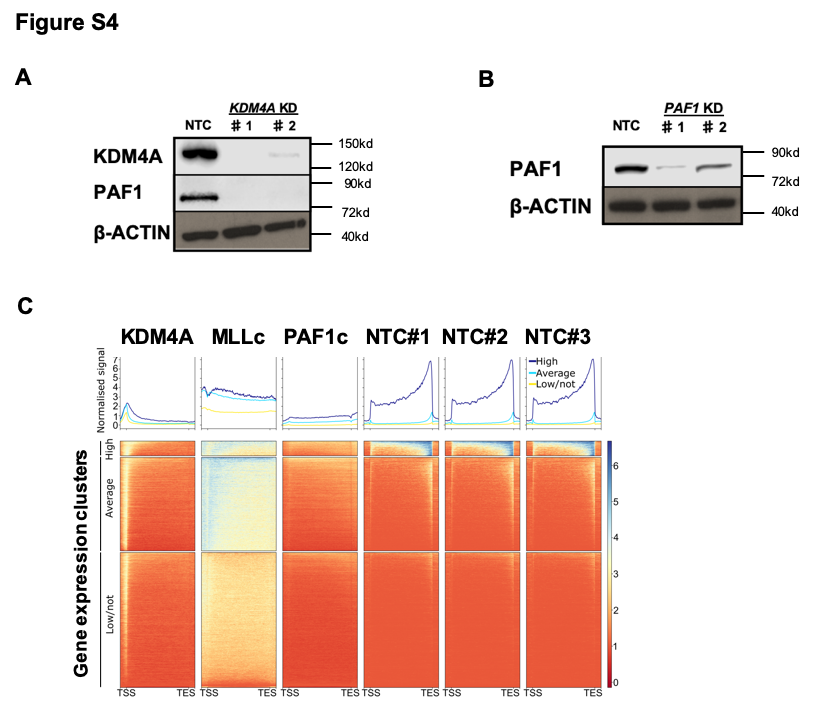


**Figure S4. KDM4A is required to maintain expression of PAF1 in human AML cells.**

The human THP1 AML cells were transduced with lentiviruses targeting *KDM4A* or *PAF1* for KD, or an NTC control, 4 days following puromycin selection. (A) Representative immunoblot showing *KDM4A* KD in THP1 cells (n = 3). (B) Representative immunoblot showing *PAF1* KD in THP1 cells (n = 3). (C) Supervised k-means clustering reveals gene expression correlates with levels of KDM4A, MLL (33), and PAF1c (PAF1) (24) at TSS. Normalised ChIP signal for KDM4A and PAF1c shown is the log_2_ ratio of read counts compared against input control. For MLL which lacks an input control normalised signal refers to RPKM. RNA-seq coverage tracks showing RPKM across all genes obtained from THP-1 cells (NTC#1, NTC#2, NTC#3) transduced with lentivirus expressing an NTC hairpin.

**
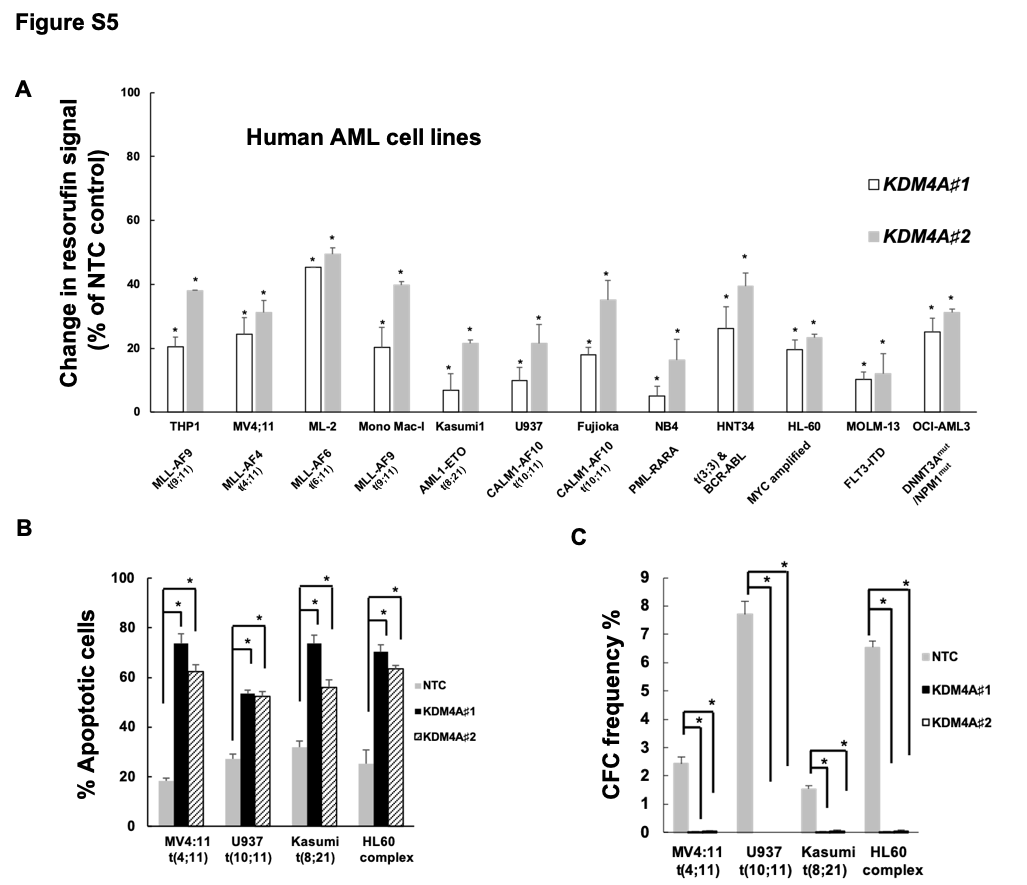
**

**Figure S5. KDM4A is required for a broad spectrum of human AML subtypes.**

(A) Resorufin signal after 4 days of *KDM4A* KD relative to NTC control cells in the indicated human AML cell lines (n=3); **p*<0.01 for comparison of each KD versus *NTC*. (B) Percentage of apoptotic cells and (C) CFC frequencies in the indicated human AML cell lines following *KDM4A* KD (n=3); **p*<0.01.

**
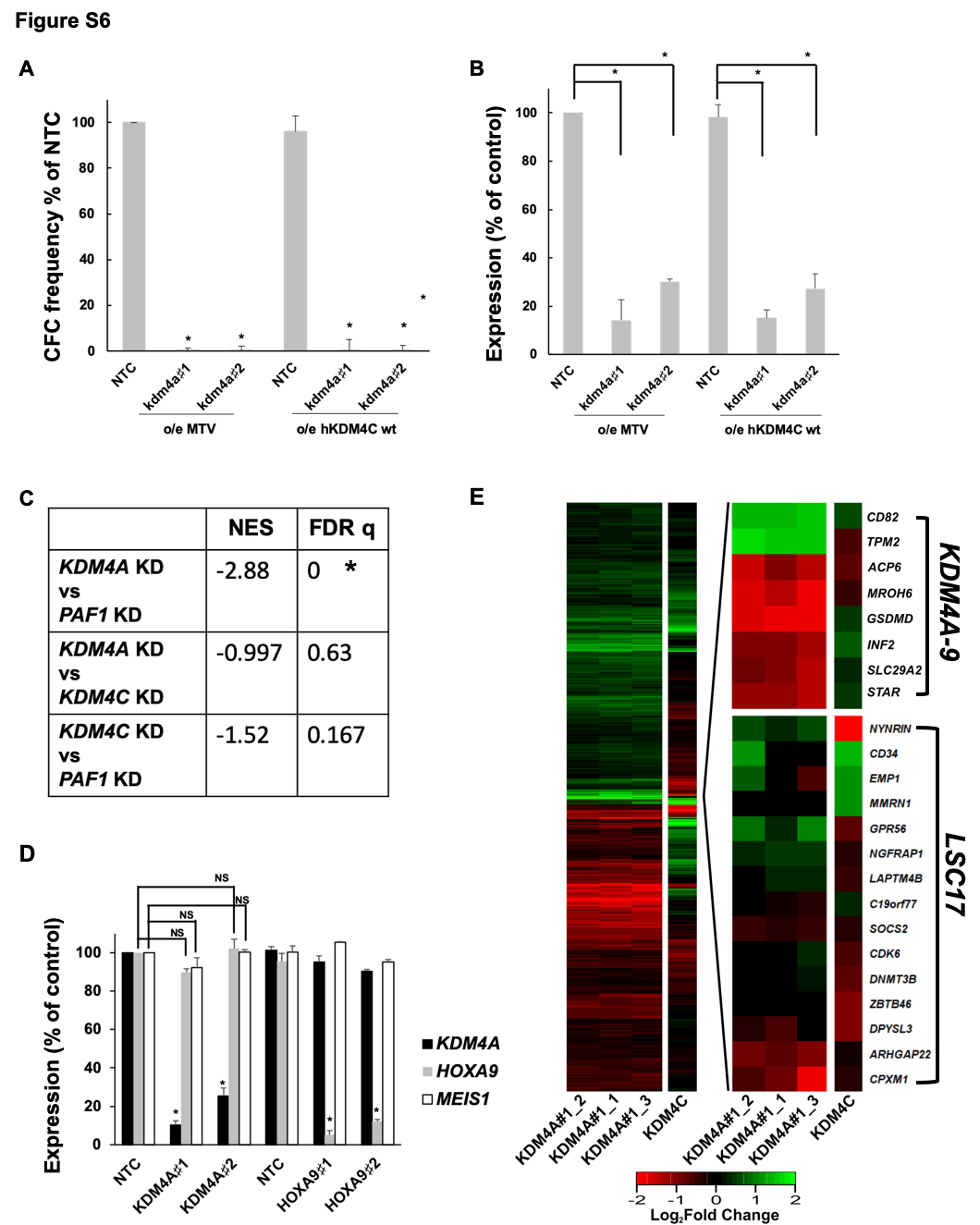
**

**Figure S6. KDM4A has a distinct function to KDM4C in AML**

(A) CFC frequencies for control and *kdm4a* KD cells from the indicated murine MLL-AF9 cells overexpressing an empty vector (MTV) or a wild type human HA tagged-KDM4C (n=3); **p*<0.001. (B) Expression of *kdm4a* in *kdm4a* KD cells from (A) relative to NTC in the indicated murine MLL-AF9 leukemic cells (n=3); **p*<0.01**.** (C) A table shows GSEA results comparing the transcriptional consequences of *KDM4A*, *PAF1* and *kdm4c* depletion, **q*=0. (D) THP1 cells were transduced with lentiviruses targeting *KDM4A* for KD, or an NTC. Bar chart showing expression of *KDM4A*, *HOXA9* and *MEIS1* determined by QPCR following *KDM4A* depletion in THP1 cells (n = 3); mean ± s.e.m. **p*< 0.0001, ^NS^*p* >0.05. (E) Heatmaps showing differences in global gene expression (padj ≤ 0.05, log_2_ FC ≥ 0.5 or log_2_ FC ≤ -0.5) after *KDM4A* and *Kdm4c* KD (left panel) and of *KDM4A-9* and *LSC17* gene signature genes (right panel) as determined by RNA-seq.
